# The healthcare experiences of middle and older age autistic women in the United Kingdom

**DOI:** 10.1177/13623613251362265

**Published:** 2025-08-12

**Authors:** Amy Gillions, Elizabeth O’Nions, Hassan Mansour, Sarah Hoare, Will Mandy, Joshua Stott

**Affiliations:** 1 University College London (UCL), UK; 2 Bradford Institute for Health Research, UK

**Keywords:** autism, autistic women, healthcare experience, older adults

## Abstract

Autistic women experience a higher prevalence of physical and mental health conditions compared with autistic men and non-autistic women, plus greater premature mortality and a higher suicide risk. However, little is known about autistic women’s experiences of healthcare, particularly in middle and later life. We aimed to explore autistic women’s perspectives on accessing healthcare services in the United Kingdom. Fifteen autistic women aged between 51 and 73 years participated in semi-structured interviews, which were analysed using reflexive thematic analysis. Four main themes were generated: (1) Stigma and stereotypes in professionals’ understanding of autism leading to poor-quality care, (2) Accumulation of negative healthcare experiences reducing participants’ confidence in services, (3) Efforts required to navigate healthcare systems and (4) The future: age-related concerns and hopes for change. The autistic women in this study reported multiple barriers to accessing healthcare and they felt stigmatised by repeated negative interactions with healthcare providers. Intersections between age and gender affected how participants felt they were perceived in healthcare settings; specifically, participants felt that being an older woman contributed to their autism-related needs being overlooked or misunderstood. Implications for services and directions for future research are discussed.

**Lay abstract:**

**Why was the study done and what did the researchers do?**

Autistic women face distinctive healthcare challenges compared with autistic men and non-autistic women. However, there is not much information about their experiences with healthcare in the UK, especially as they age. To better understand the lived experiences of this population, the research team in this study interviewed 15 autistic women in middle to later life about their healthcare experiences. Information from the interviews was analysed by creating themes based on what the participants had reported.

**What did the researchers find?**

Four main themes came out of the analysis.

1. Participants expressed concerns that stigma and stereotypes associated with autism led to poor healthcare experiences.

2. Participants’ confidence in seeking help was affected by having many negative experiences across their lives. Difficult interactions with healthcare providers also made them less confident in seeking help.

3. Navigating the healthcare system was a challenge for participants.

4. Participants thought a lot about the future. They had worries about ageing and what consequences this might have on their health and support needs. They also shared hopes for better healthcare in the future.

**What do the findings mean?**

The findings highlight that autistic women in middle to later life face many barriers in accessing healthcare. The study emphasises the need for better understanding and support for autistic women in healthcare settings.

## Introduction

Autism spectrum disorder (hereafter ‘autism’) is a neurodevelopmental condition diagnosed on the basis of repetitive and restricted behaviours and difficulties with social interaction and communication (American Psychiatric Association, 2013). Epidemiological studies suggest that at least 1% of people are autistic (Brugha et al., 2016) although estimates vary up to 3% depending on the age demographic (Shaw et al., 2025), and the ratio for male to female diagnosis is estimated to be 3:1 from community-based studies (Posserud et al., 2021).

Autism is a lifelong condition, although it is most often diagnosed in childhood. Most autism research, however, has concentrated on autism in childhood and young adulthood and only 1% of publications about autism focus on older age (Mason et al., 2022). Across the lifespan, females are underrepresented in autism research, meaning that there is less known about experiences and needs of autistic women entering middle and later life (D’Mello et al., 2022). Given the increased prevalence of health needs with age, research focused on middle-aged and older autistic adults and their health and support needs is particularly important (O’Nions et al., 2024).

### Autism and health

Compared with the general population, diagnosed autistic people have a higher prevalence of physical and mental health conditions across the lifespan (Croen et al., 2015; Lever & Geurts, 2016) and are more likely to have a learning disability (Russell et al., 2019) or attention-deficit hyperactivity disorder (Hours et al., 2022). Gastrointestinal issues, sleep difficulties, epilepsy, depression, and anxiety are among the most common co-occurring conditions experienced by autistic people (Fortuna et al., 2016). Autistic people also have a lower overall life expectancy (O’Nions et al., 2024) and a higher risk of death by suicide (Hirvikoski et al., 2020) compared with neurotypical adults, reflecting health inequalities and unmet support needs.

### Autistic women’s health

Autistic women appear to have poorer health status compared with autistic men. They experience a higher prevalence of certain health conditions such as epilepsy and genetic disorders, and show disproportionately poorer health compared with non-autistic women (Kassee et al., 2020). Mortality risk for autistic women is also greater than for autistic men (Hirvikoski et al., 2016) and autistic women without intellectual disabilities present with increased risk for death by suicide compared with men (Kõlves et al., 2021). Women’s health issues, including the impact of the menstrual cycle and menopause, are also known to have a greater impact relative to non-autistic women (Groenman et al., 2022).

In addition, late diagnosis of autism is more common in women than men and may contribute to poorer health outcomes. For example, autistic women are more likely to be referred to mental health services for support with conditions such as anxiety and eating disorders, reflecting gendered assumptions in healthcare pathways (Tint et al., 2017).

Many autistic women are also misdiagnosed with personality disorder which further contributes to the unsuitability of health services created by barriers associated with gender and delayed identification of autism (DaWalt et al., 2021). The intersection of gender and autism may therefore amplify health disparities, where autistic women are simultaneously navigating ableism and sexism within healthcare systems (Botha & Frost, 2020).

### Healthcare experiences of autistic people

Despite the complexity of their health needs, autistic people’s experience of healthcare has been little studied. Those findings that do exist suggest autistic people, and autistic women in particular (Babb et al., 2021; Hampton et al., 2022), have significant difficulties accessing healthcare (Weir et al., 2022), resulting in health inequalities and a high level of unmet healthcare needs in the autistic population, for both physical and mental health conditions (Płatos & Pisula, 2019). Autistic people also report lower satisfaction with healthcare (Gerber et al., 2017) and higher utilisation of services compared with non-autistic people (Zerbo et al., 2019).

Barriers to healthcare access exist for autistic people at the individual, service provider, and systemic level (Walsh et al., 2020). These barriers include insufficient knowledge about autism among healthcare professionals leading to adverse experiences with providers (Shaw et al., 2024). Furthermore, many autistic people report heightened sensory sensitivities in healthcare environments (e.g. lighting, noise, invasive procedures) which cause distress and avoidance (Babalola et al., 2024). Communication differences associated with the ‘double-empathy problem’ can also lead to challenging interactions with professionals when attempting to access support (Strömberg et al., 2022).

While these studies provide valuable insights, it is important to recognise that samples in studies examining the experiences of autistic women in healthcare and beyond predominantly comprise autistic individuals in childhood, adolescence or early adulthood. For example, Walsh et al. (2020) and Stromberg et al. (2022) focus largely on younger cohorts, and similarly, Shaw et al. (2024) and Babalola et al. (2024) draw from populations skewed towards early to mid-adulthood. There remains a paucity of research focusing on the healthcare experiences of autistic individuals in middle and older adulthood, particularly autistic women.

### Current study

Despite the increased need for healthcare services with age and the evidence for the particular health needs of autistic women, there are no studies, to our knowledge, which focus specifically on the healthcare experiences of middle and older age autistic women. Furthermore, health and ageing research has been highlighted as a priority for the autistic community (Michael, 2015). The aim of this study was to qualitatively explore autistic women’s healthcare experiences in the United Kingdom, keeping the scope purposefully broad and exploratory. In the United Kingdom, healthcare is primarily provided through the National Health Service (NHS), a publicly funded system offering universal healthcare that is free at the point of access. A qualitative approach was chosen to investigate the following research questions: (1) ‘How do middle and older age autistic women perceive support from healthcare services?’ and (2) ‘What are the barriers and facilitators reported by middle and older age autistic women when it comes to finding and accessing healthcare services?’.

## Methods

### Participants

Fifteen participants were purposively sampled (for age, ethnicity and sexuality) between June 2022 and December 2022 as a subsample of those taking part in a larger survey asking autistic people about their healthcare experiences, which was advertised via posters put on social media (X, Facebook), pages of autism researchers and through the mailing list of charities for autistic people (UK’s National Autistic Society, Autistica). To be eligible, participants were required to be (1) female (either assigned female sex at birth or currently identifying as a female); (2) aged 50+; (3) diagnosed autistic or self-identifying as autistic; (4) able to hold a conversation in English (this was established through communicating with participants to arrange the interview); and (5) a UK resident. Potential participants were excluded if they had multiple and profound intellectual disabilities that may prevent participation in an interview and capacity to consent.

Participants were all aged between 51 and 73 years and further demographics are presented in Supplementary Table 1. A formal diagnosis of autism was not necessary to take part as there are known barriers to assessment and diagnosis in adulthood, particularly for women. The Autism Quotient‒10 (AQ-10) was used to quantify autistic traits and the median score for participants was eight. The AQ-10 is a brief tool designed to indicate if a person has a high presence of autistic traits, with a score of six indicating possible autism (Allison et al., 2012).

### Participatory methods and community involvement statement

A steering group of four autistic adults aged 50+, including three females and one male, were involved in the development of the overall research project and all study materials. This included the online survey and interview topic guide where they offered suggested revisions to improve clarity, accessibility and relevance to lived experiences. They also provided feedback on the practical aspects of completing the survey (e.g. timings, clarity of information and comprehensiveness). Their feedback led to concrete changes in both the content and structure of study materials, ensuring they were more inclusive and representative.

### Materials and procedure

A topic guide for the interviews was developed by the research team in collaboration with the steering group of older autistic people (please see Supplemental Material). Interview questions were developed from selected topics based on findings from existing research focusing on barriers and facilitators to healthcare in autistic people, alongside broader questions about gender and ageing related to being autistic. Interview schedules were piloted with the steering group and subsequent adaptations were made based on feedback such as including specific prompts, providing definitions, and simplifying language. The interview schedule was used flexibly, allowing the interviewer to intuitively follow the conversation while having a basic framework to guide the discussion.

Individuals who gave written informed consent to participate in an interview subsequent to their completion of the survey, and who were identified as eligible, were interviewed either on the telephone or online via Zoom, with reasonable adjustments offered. All interviews were recorded on an encrypted voice recording device. The first author transcribed all 15 audio files verbatim, removing any identifying information. Anonymised survey data were then linked with a transcript through the allocation of a participant number. Participants each received a £15 voucher for taking part in the interview.

### Data analysis

Reflexive thematic analysis (RTA; Braun & Clarke, 2019) was used to analyse qualitative interview data. Before embarking on the analysis, a bracketing interview was completed with another member of the research team to position the author’s identity and pre-existing assumptions about the topic. A position statement was written based on this interview.

Following analysis guidelines (Braun & Clarke, 2006), interviews were transcribed, read and reread by the first author then coded using N-Vivo. Coding was conducted at a semantic level, emphasising the explicit content of participants’ language rather than interpreting underlying meaning and drew on a ‘bottom-up’ inductive data-driven approach to capture meaning as directly communicated by participants using an experiential orientation (Braun & Clarke, 2013). Two of the coded transcripts were shown to another member of the research team to discuss coding ideas and explore assumptions made about the data to enhance the richness of the coding rather than checking for consistency, as advocated by Braun and Clarke (2021).

Clusters of codes were generated through looking at individual codes and mapping them together into thematic ideas with co-authors. For example, if a participant described finding noise in hospitals challenging, this was coded as ‘sensory sensitivities’ and clustered together into a thematic idea such as ‘environmental barriers’. These clusters were then used to create a set of initial themes, which were developed further using a thematic map. Final themes, which are explored in the results, were named and refined through discussion with the research team.

### Data availability statement

The participants of this study did not give written consent for their data to be shared publicly, so due to the sensitive nature of the research supporting data is not available.

## Results

### Theme 1: Stigma and stereotypes in professionals’ understanding of autism leading to poor-quality care

Participants reported that healthcare experiences were significantly impacted by professionals’ understanding of autism. Repeated encounters with professionals who held stereotypical views of autism made participants feel stigmatised and resulted in poor-quality experiences of care: ‘*The level of knowledge amongst health professionals about being autistic and what that means is extremely poor and usually results in them being absolutely patronising and treating you like you’re some sort of idiot*’ (P15).

Participants commented on the lack of understanding among healthcare professionals about female autism presentations and reliance on stereotypes informed by autistic males:

*They seem to have an idea about what autism is and isn’t . . . and still think maybe that women can’t be autistic . . . so it’s actually at a point where I’ve decided not to even bother saying anything about it anymore*. (P11)

They described having their diagnosis questioned because their interaction with healthcare professionals was incongruent with stereotypically male autistic behaviour: ‘*They were saying, oh, she can’t be autistic ‘cause she can do this and she can do that*’ (P8). Participants were left feeling discouraged and unsure about whether to inform services that they are autistic. For those who did, they raised concerns about having to educate professionals to receive better support: ‘*My most recent counselling support person recognised she didn’t know anything about autism . . . she was prepared to go away and read and learn but I felt like I was having to support somebody to support me*’ (P9).

Perceived professional stereotypes affected participants’ experiences of receiving mental health care in particular. Gendered stereotypes were perceived to influence professionals’ perceptions of their mental health, which was perceived to affect the treatment and support they were offered. Participants described routine misdiagnosis of autistic traits as mental illnesses, ‘*You go to the GP, they put it down to anxiety or depression . . . they were manifestations of undiagnosed autism*’ (P6), and the experience of feeling let down by healthcare professionals who had been unable to identify they were autistic: ‘*I’ve had an awful lot of counselling-type therapy over the last 25 years, not one of them picked up that I was autistic*’ (P14).

Participants described receiving support for their mental health difficulties which was ineffective due to lack of adaptations for their autism-related needs: ‘*There’s nothing really that’s particularly tailored for autistic people and I haven’t found CBT delivered in the standard manner very helpful*’ (P10). Lack of adaptations led participants to assume something was wrong with them and their autistic styles of thinking, rather than the treatment being unsuitable:

*Where you’re being supported for anxiety and depression, you are led to believe that your way of thinking is wrong . . . and there’s something very wrong about you. And actually, that’s just not true. I just need to be supported differently*. (P9)

Participants also reflected on the consequences of not having had autism recognised and being misdiagnosed with mental illnesses for most of their adult lives. Repeatedly accessing different supports in the hope that something would help led to frustration and sadness. Many participants blamed themselves for problems finding support, rather than the lack of services tailored to their needs:

From my 20s on I’ve tried f***ing everything under the sun, any kind of random weird little therapy you’ve ever heard of, I’ve probably tried it . . . you then end up in your 50s and think, so this is never going to go away then? (P15)

Interacting with dismissive professionals who lacked understanding about autism led participants to feel mistreated: ‘*As an autistic person, I don’t want to have to do this because I know it will be a confrontation. I know it will because you, the doctor, doesn’t understand my perspective at all*’ (P14). Participants felt that being autistic, and specifically an autistic woman, made it more likely professionals would not believe them, minimise their concerns and treat them poorly: ‘*I’m not listened to, I’m not understood. . . . They dismiss what I’m saying . . . then they try to tell you what you’re saying isn’t true*’ (P11).

Further consequences of poor-quality care associated with stigma and stereotypes meant that participants often felt overlooked, judged or dismissed due to their status as middle or older-aged autistic women: ‘*I’ve been judged as a parent, I’ve been judged as a woman, as a middle-aged woman, as an autistic woman . . . I’m looking all the time for ways to make sure I’m not being misjudged*’ (P13). Being a woman within a family also impacted participants’ ability to prioritise their own health needs: ‘*The age 50 group, you’ve got your parents as well . . . your own health is like right down the bottom of the list*’ (P3). One participant reflected on how her late diagnosis of autism impacted her interactions with professionals about her autistic children: ‘*As an autistic woman, I’m trying to get help for my autistic children. I didn’t know I was autistic, so I found that really difficult, and my daughters have never really had the right sort of help*’ (P3).

### Theme 2: Accumulation of negative healthcare experiences reducing participants’ confidence in services

Many participants described having complex healthcare needs and needing to attend many appointments. An accumulation of bad experiences created negative anticipation about accessing healthcare: ‘*So you might get through one appointment and what happens if that’s a bad appointment? Then you’ve got to go for another one, and then another one . . . you feel like you’ve got to suffer on your own*’ (P2). Positive healthcare experiences were rarely reported but when they occurred, they triggered surprise or confusion: ‘*When I do come across someone that does have that element of empathy or humanity, it’s jarring because it’s unusual*’ (P12).

Participants felt so disillusioned with healthcare services that they did not want to bother seeking help anymore, questioning whether it was worth putting themselves through difficult encounters: ‘*If you have enough things go wrong, enough let-downs, enough bad experiences, you just think it’s really not worth it for like a small niggle*’ (P3). Lack of appropriate care fostered resentment, bitterness, and frustration and reduced participants’ confidence in the ability of service providers to offer appropriate support: ‘*Often I’ll go back for the same thing over and over again because I’m not getting any help. And then, in the end, I just think what’s the point?*’ (P7).

Some participants believed that they were problematic users of the system who were burdening service providers by trying to access healthcare; ‘*There are times when I think to myself, I can’t bother them again. I’m going to be perceived as a pain*’ (P12), and picked up on negative cues from providers: ‘*The pharmacy seem to know my name as soon as I walk in, I don’t feel like it’s friendly, I just think they feel “oh no, it’s her again*’ (P2). Repeated experiences like this meant some participants felt unworthy of support as a result.

Difficulties tolerating distressing sensory environments contributed significantly to negative healthcare experiences: ‘*You’re left in environments where you feel unsafe, it’s crowded, there’s no attempt to make any reasonable adjustments*’ (P11). Participants’ sensory sensitivities, especially to pain and touch, were a barrier to accessing routine women’s health screening: ‘*I’m afraid I never went for [cervical] smears, it just felt too much*’ (P10). Participants believed that avoiding significant sensory distress outweighed the potential benefits to health, although some had concerns: ‘*Mammograms I found especially painful. I’ve only had one, I didn’t go to my last one. And I’m worried about my health and I know I’m at the age where these things need to be explored*’ (P9).

Feeling continually let down by services led participants to feel overwhelmed and anxious about engaging with them: ‘*It’s just hell. I feel anxious and stressed before I go. It always turns into a bad experience so I’m in a heightened state of alert*’ (P11). Participants also described significant distress during healthcare encounters: ‘*I can feel like waves fear coming up through the body, it’s like pulsing. You know, that’s why I can’t think straight when I’m talking to the GP*’ (P7). The repeated negative encounters across their lives were experienced by some participants as traumatic.

### Theme 3: Efforts required to navigate healthcare systems

Participants felt that an enormous effort was required of them to navigate healthcare systems. Several participants equated interactions to a ‘fight’ or ‘battle’: ‘*I have it in my mind that no matter what I go to the GP for, I’m going to have to fight to be heard*’ (P14). The persistence and perseverance required left participants feeling uncared for. This was compounded by difficulty accessing an autism diagnostic assessment: ‘*The diagnostic quest that’s been ongoing since my 40s to try and get answers to these things has probably contributed to my burnout because it’s been so fundamentally exhausting*’ (P6).

Participants described masking or concealing their autism-related difficulties. Generally, participants wanted to present to services as compliant to avoid being perceived as a burden: ‘*I don’t want to be a problem, I don’t want you to know if I’m worried. I try really hard to cover that all up and I don’t want to be a nuisance*’ (P9). For some, masking enabled them to cope in appointments and access care. For others, it concealed the extent of their difficulties and meant they were offered inadequate or unsuitable care. Some participants expressed concern over lack of awareness about masking: ‘*It would be quite nice for healthcare professionals to be aware of how capable autistic women are of coming across as neurotypical if we’ve got the energy*’ (P13).

Organising oneself to attend an appointment, and using memory, planning, and communication skills to describe a health problem required significant effort from participants. Participants reported that the practical difficulties of getting to appointments (e.g. parking, travel, timings) and the cognitive aspects (e.g. planning, memory) both had a negative impact on their ability to engage effectively: ‘*It might only be like a 20 minute or 10 minute appointment, but that is my whole day’s activity gone ‘cause I’ve got no energy left for anything else*’ (P4).

### Theme 4: The future: age-related concerns and hopes for change

Participants were worried about how a system that currently does not meet their needs could provide adequate care for them in later stages of life: ‘*It worries me with the state of the NHS how that is going to be moving forward as I get older. I worry about the impacts of age on my existing conditions and my autistic sensitivities*’ (P6). Negative experiences of care for minor complaints led to anxiety about more serious conditions and hospitalisation:

*If I had to be on a ward for a week, I would really really struggle, and that is when the mask would come off, that’s when they would see me unravelling and I would worry very much about how I would be treated*. (P9)

Reflecting on the future left many participants feeling extremely let down by the healthcare system and scared about what might happen to them. Some participants reflected on the likelihood that residential care homes might not be suitable for them: ‘*If an autistic person were put in a care home, I think they would have particular needs and I hope I don’t have to go near one*’ (P5).

Alongside negative healthcare experiences, participants also described some examples of good practice, which offered some hope. The introduction of telemedicine during the pandemic offered participants hope that flexible healthcare was feasible, fostering more choice for appointments and acceptability of different ways of communicating with healthcare professionals: ‘*To have the option of an online appointment is a good thing for me, and so there was some improvements. There’s a bit more flexibility*’ (P7). Participants suggested that these practical adjustments make the likelihood of positive healthcare experiences more of a possibility.

Participants showed an empowered stance in advocating for the healthcare that they deserve by making clear suggestions for ways forward. Hopes for change were positioned around an individualised approach to healthcare for autistic people: ‘*Healthcare professionals need to treat us and talk to us as individuals. There might be some common themes but we’re all very different*’ (P9). Participants highlighted the importance of healthcare professionals asking individuals about their specific needs, avoiding making assumptions and offering appropriate adjustments using person-centred care:

*I’d like healthcare professionals to know that everybody who’s autistic is different. And to just say, how does it affect you? And know that it might affect you in positive ways as well as negative ways. Or ask, what’s the best way for us to communicate? It would be useful just for people to say, what does being autistic mean for you?* (P13)

Areas for service improvement identified by participants included facilitating communication preferences, providing clear written information prior to and after appointments, asking about and accommodating sensory needs, making health screening appointments more autism-friendly, providing mandatory autism training to professionals, and proactively asking individuals if they require adjustments.

## Discussion

This was, to our knowledge, the first study to look at the healthcare experiences of middle and older age autistic women in the United Kingdom. The four themes identified pointed to a sense of stigma and misunderstanding, which was compounded by the intersectionality of female gender, autism and ageing leading to cumulative negative healthcare experiences. Difficulties navigating the healthcare system were highlighted alongside hopes for change in the future coupled with fears related to ageing.

### Connecting themes to wider literature

The first theme of stigma and misunderstanding fits with existing research about autistic adults’ experiences of healthcare (Calleja et al., 2022; Mason et al., 2021; Mazurek et al., 2023) and lack of autism knowledge among healthcare professionals (Walsh et al., 2020). We highlight additional concerns from autistic women about professionals’ specific lack of knowledge around the female autism phenotype and the impact of this on professionals’ views about their diagnosis and experiences of missed opportunities where autism could have been identified. Formally diagnosed participants felt that the accuracy of their diagnosis was questioned by professionals resulting in dismissal of their health concerns and neurodivergence not being accommodated as part of their care.

Consistent with findings regarding the misattribution of autism-related behaviours in women to mental health difficulties or personality disorders (Kentrou et al., 2021), participants shared their journeys of struggling to understand their mental health needs throughout adulthood in the absence of an autism diagnosis leading to a lack of appropriate support. This experience is known to contribute to poorer mental health outcomes for autistic women (Beck et al., 2020), and findings from our study build on previous work to suggest that this problem may become magnified over time with ageing.

Our second theme supported and added to research detailing younger autistic adults’ negative healthcare experiences (Gerber et al., 2017). Our findings indicate that, with advancing age, negative experiences accumulated, leaving participants feeling resigned and avoidant in seeking healthcare support. In particular, findings from this study suggest that middle and older autistic women experience intense emotional distress when engaging with healthcare, something that needs urgent investigation in larger, more representative samples.

Our findings also affirmed the relevance of known barriers to healthcare for autistic adults such as sensory sensitivities (Calleja et al., 2022; Walsh et al., 2020) is also relevant for middle and older age autistic women. This study extended this understanding by highlighting gendered aspects of sensory challenges. Notably, participants described sensory difficulties with medical procedures such as cervical smears and mammograms. This was a barrier for participants to attend preventive health screening, with some women in the study never having attended these appointments. This reinforces the importance of appropriately adapted care to ensure equity in access to preventive services.

In this study, participants reflected on a lifetime of masking and assessed both the costs and benefits of masking during interactions with health professionals. Given that little is known about older autistic people’s experiences of masking (Sonido et al., 2020), this study offers some early insights into how autistic women of this demographic describe masking in the context of healthcare, and suggests that it represents an important coping strategy, but one that could mean the true extent of a person’s challenges remains invisible to providers.

Participants had many suggestions about facilitators to good healthcare, but similar to other studies, rarely experienced them in practice (Mazurek et al., 2023). There was a clear distinction between the desired expectations for healthcare and reality, although some reported positive healthcare experiences offer clear directions for services to make small adjustments that may make a significant difference in how autistic women perceive support (e.g. offering different appointment modalities). The similarity between suggestions in this study and those in the wider adult autistic population, such as offering flexibility and individualised care (Adams & Young, 2021; Mason et al., 2019; Nicolaidis et al., 2015), suggests there are many factors which facilitate healthcare that are relevant across gender and lifespan and are currently lacking in services. Such facilitators may be especially important for autistic adults in middle and older age who may be more likely to use healthcare services due to advancing health needs with age (Mukaetova-Ladinska et al., 2012).

### Intersectionality: age, disability and gender

Older autistic women may experience greater stress resulting from discrimination, oppression and societal prejudice (Meyer, 2003), due to their multiple marginalised (women, older) and minority (autistic) statuses. Participants themselves highlighted the importance of talking about their experiences from the position of intersectionality, and how the convergence of the different aspects of their identity influenced their experiences of healthcare and how they were perceived by professionals. Therefore, the individual and collective impact of possible sexism, ageism and ableism experienced by middle and older age autistic women is likely to influence their healthcare experiences. Healthcare systems designed predominantly around neurotypical, male, and younger norms risk excluding middle and older age autistic women who sit at multiple marginalised intersections (Botha & Frost, 2020).

Findings appear to fit the ‘triple empathy problem’ developed from a recent study which found that autistic people experienced communication difficulties with healthcare professionals due to mutual misunderstandings rather than deficits in autistic people (Shaw et al., 2024). The authors argue that in healthcare settings, these communication difficulties are exacerbated due to differences in culture and agendas of professionals which increase relational challenges (Shaw et al., 2024). Our findings illustrate the additional challenges experienced by middle and older aged autistic women, where these communication gaps are magnified not only by neurodivergence but also by gendered expectations around communication and emotional expression, and by age-related stereotypes.

### Strengths and limitations

Strengths of this study include the breadth of the interview schedule, meaning that it makes a broad contribution to a nascent area of research. Limitations include the lack of racial and ethnic diversity in the sample (all but one participant was White). Racial and ethnic minoritisation is known to adversely affect health outcomes and experience of healthcare services (Marmot, 2017), so it is particularly important for future work to gather those voices, particularly in light of the intersectional issues raised in this study. Participants in this study were recruited online and thus had access to, and were literate in, technology. Participants experiencing socioeconomic deprivation and/or health needs that prevented internet use or active engagement with technology were thus not represented. This impacts the generalisability of these findings. While internet usage in older adults is increasing significantly (Zhang et al., 2021) and there is some evidence autistic people prefer computer-mediated communications (Westerberg et al., 2021), it is unclear whether autistic adults might disproportionately experience digital exclusion. Future work should use offline recruitment methods to ensure inclusivity.

Only two participants identified as having a mild learning disability were included. This is especially important as a limitation given that individuals with co-occurring autism and intellectual disability experience greater inequalities and premature mortality (Krantz et al., 2023). The majority of participants were also educated to degree level so findings may be biased towards an intellectually able and affluent demographic which is likely to favourably impact healthcare experiences.

Information about autism diagnosis was based on self-report only, and participants who self-identified as autistic but had not been formally diagnosed were also included. While it is recognised that this may create issues with credibility and validity, there are many justifications for this. Notably, many older autistic people may choose not to pursue a diagnosis and women in particular face several barriers (Lewis, 2017) or have difficulty obtaining historic medical records if they were diagnosed in childhood. A recent paper examining the underdiagnosis of autism in the United Kingdom highlighted that fewer than one in 10 autistic adults above 50 were diagnosed based on primary care records as of 2018 (O’Nions et al., 2023). Therefore, limiting the sample to only diagnosed people could mean that the sample would have been unrepresentative. The panel of autistic people involved in the design of the study also felt it was important that self-identified autistic people were eligible to participate and our goal as a research team was to reduce barriers to participation in a vastly under-researched population.

### Clinical implications

These findings indicate that there are important health inequalities in the United Kingdom that adversely impact the health and well-being of older autistic women. While it is important not to overgeneralise or overstate findings from a small qualitative study such as this, given the extensive health inequalities experienced by middle and older age autistic women, these findings should motivate further investigation into their experiences.

The findings from this study are highly relevant to current legislation and the development of health services. For example, the National Autism Strategy in the United Kingdom (HM Government, 2021) highlighted the need for autistic voices to be heard to improve healthcare access for autistic people. A clear implication of this study is the need for high-quality training for professionals about autism, including specific content about gender and ageing. Involving autistic people in the development and facilitation of training as in the UK’s recent mandatory training on learning disability and autism should also be a priority (NHS England, 2022).

Findings suggest reasonable adjustments that are individualised and person-centred should be readily offered, and the onus should not always be on the patient to make these requests (Brice et al., 2021). The goal of these adjustments should be to reduce cognitive load and emotional distress for autistic women when engaging with healthcare. Some examples include choice of appointment type, offering extended appointments, and accepting different communication methods.

There are further implications at a broader UK service-provider level. First, consideration of sensory sensitivities for gendered healthcare screening should be prioritised to make it easier for autistic women to attend such appointments. This should be considered in conjunction with broader service-wide adjustments to cater for sensory needs across the autistic population, such as offering people a separate place to wait for appointments (Strömberg et al., 2022).

Additionally, for adult women with multiple chronic physical health conditions and ongoing mental health difficulties, services could consider screening for autism as part of routine healthcare (Kassee et al., 2020). This would likely contribute to earlier diagnosis of autism in women and enable their neurodivergence to be incorporated into their physical and mental health treatment plans. A good example of this in current healthcare practice has been demonstrated by a female eating disorder inpatient ward where patients complete an autism screening measure upon admission (Tchanturia et al., 2020).

### Directions for future research

Future studies should seek to include the views of autistic women from racially and ethnically minoritised backgrounds in the UK - also referred to as the global majority - to better understand their experiences. Studies may benefit from explicitly applying health minority approaches to better understand how intersecting identities shape healthcare access and outcomes. They should also employ quantitative approaches using population-representative samples to identify how widespread specific types of barriers to healthcare are among autistic women, to identify priority areas for redesign of services. At present, little is known about the healthcare experiences of middle and older age autistic women with co-occurring intellectual disabilities or the views of carers (Mason et al., 2022), which is an important area for further research.

More broadly, there is limited research about specific gendered healthcare experiences in autistic women. Autistic people point to the importance of research regarding menopause, hormone-replacement therapy, and experience of appointments such as mammograms (Michael, 2015). While this study was purposefully broad, further research is needed to create an in-depth understanding of how middle and older age autistic women experience specific types of healthcare and differences compared with neurotypical women. This work also demonstrates the importance of further research about health and social care provision for autistic women in older age with physical health decline. For example, research should consider how older autistic women perceive healthcare in residential settings (Rodgers et al., 2019).

Finally, more research is needed from the perspective of service providers and professionals. Specific studies focusing on healthcare professionals’ understanding of autism in women are required to identify gaps in knowledge and assess provider confidence. The issues with mental health misdiagnosis and ineffective treatment identified in this study indicate the need for research exploring how mental health professionals distinguish autism from mental health conditions in women to reduce delayed diagnosis or, conversely, diagnostic overshadowing (Bhargava & Ashwin, 2025).

### Summary

This work provides an in-depth understanding of the healthcare experiences of autistic women in middle and older age. This is the first study to explore the perspectives of this population. Further research is required to address the significant unmet healthcare needs and healthcare dissatisfaction among autistic women. Access to effective healthcare and feeling heard and validated by healthcare professionals is of paramount importance in improving healthcare experiences in the United Kingdom and reducing health inequalities for autistic women across the lifespan.

## Supplemental Material

sj-docx-1-aut-10.1177_13623613251362265 – Supplemental material for The healthcare experiences of middle and older age autistic women in the United Kingdom Supplemental material, sj-docx-1-aut-10.1177_13623613251362265 for The healthcare experiences of middle and older age autistic women in the United Kingdom by Amy Gillions, Elizabeth O’Nions, Hassan Mansour, Sarah Hoare, Will Mandy and Joshua Stott in Autism
