# Supplementary material for: The healthcare experiences of middle and older age autistic women in the United Kingdom
Source: Autism. 2025 Aug 12;30(1):49–60. doi: 10.1177/13623613251362265 (PMC12717282; doi:10.1177/13623613251362265)
Supplement: sj-docx-1-aut-10.1177_13623613251362265 – Supplemental material for The healthcare experiences of middle and older age autistic women in the United Kingdom [file sj-docx-1-aut-10.1177_13623613251362265.docx]

**The Healthcare Experiences of Middle and Older Age Autistic Women in the UK**

**Supplementary Document**

**Table 1.**

*Participant Demographic Information.*

| **Demographics** | **N (%)** |
| --- | --- |
| **Gender** | |
| Female (assigned at birth) | 15 (100%) |
| **Autism Diagnosis** | |
| Formal diagnosis | 12 (75%) |
| Self-identifying | 3 (25%) |
| **Intellectual Disability** | |
| None | 13 (86.6%) |
| Mild | 1 (6.6%) |
| Self-identified learning needs | 1 (6.6%) |
| **Ethnicity** | |
| White British | 14 (93.3%) |
| Mixed or Multiple: White and Asian | 1 (6.6%) |
| **Sexual Orientation** | |
| Heterosexual | 12 (75%) |
| Prefer not to say | 2 (13.3%) |
| Asexual | 1 (6.6%) |
| **Living Status** | |
| With partner/spouse | 10 (66.6%) |
| Alone | 5 (33.3%) |
| **Education** | |
| Up to postgraduate level | 6 (50%) |
| Up to undergraduate level | 5 (33.3%) |
| Did not complete | 2 (13.3%) |
| School to age 18 | 1 (6.6%) |
| School to age 16 | 1 (6.6%) |
| **Employment Status** | |
| Retired | 5 (33.3%) |
| Employed (part-time) | 2 (13.3%) |
| Employed (full-time) | 4 (26.6%) |
| Partially retired | 1 (6.6%) |
| Volunteer | 1 (6.6%) |
| Not employed | 2 (13.3%) |

**Semi-Structured Interview Schedule**

Healthcare needs:

- What are your current healthcare needs?
  - Do you have a diagnosis for any physical or mental health difficulties?
  - Have you received a diagnosis for a condition which you felt was inaccurate?
  - Do you have any current concerns about your healthcare needs?
- If you have started the menopause, has this affected your healthcare needs?
- What have been your healthcare needs been in the past?

Access to services:

- How often do you access healthcare services, if at all? (this can include GPs, pharmacies, dentists, chiropodists, community or hospital services, outpatient, nursing, opticians, mental health services, occupational therapy etc.)
- What helps when trying to access, or engage with healthcare services?
- How easy is it to access support for any diagnoses you have?
- What is it like contacting your GP?
- Have you been to hospital in the last few years?
  - If yes, what was this experience like?
  - Have you heard of the autism hospital passport? If so, would you consider using it or have you ever used it?
- Does anyone support you to attend healthcare appointments?

Experience of services:

- What is your experience of services that offer healthcare support to you?
  - How do you feel you are treated?
  - Do you feel like they listen or understand?
  - Do you feel respected?

Ageing:

- How have your healthcare needs changed, particularly as you have gotten older?
- How do you think your experience of services or accessing services compares now to how it was when you were younger?

Gender:

- What is your experience of appointments specifically related to women’s health (e.g. cervical smear, mammogram)?
- Is there anything you want to say about your experiences as an autistic woman accessing healthcare services?

Facilitators, and barriers to healthcare services:

- Have you experienced any difficulties when trying to access or engage with services? (this can include GPs, pharmacies, dentists, chiropodists, community or hospital services, outpatient, nursing, opticians, mental health services, occupational therapy etc.)
- What are the barriers which prevent you from accessing healthcare services?
- Has anything helped you overcome these barriers in the past?
- How do other aspects of your identity/life influence your healthcare needs and access to services? (e.g. ethnicity, religion, gender, sexuality, socioeconomic status, spirituality, appearance, or your wider social network including family, friends, and support groups)
- Have services tried to adapt to meet your needs?
- What helps facilitate access to healthcare services, particularly in middle/older age?
- What impact has the pandemic had on your experiences with healthcare services?

Summing up:

- Do you feel that being autistic affects your healthcare needs? If so, how?
- Do you feel like being autistic affects your experiences of healthcare services? If so, how?
- Is there anything else we should be asking autistic women?
- How could healthcare services be improved for middle-aged/older autistic people?
